# Methotrexate Treatment of Newly Diagnosed RA Patients Is Associated With DNA Methylation Differences at Genes Relevant for Disease Pathogenesis and Pharmacological Action

**DOI:** 10.3389/fimmu.2021.713611

**Published:** 2021-11-18

**Authors:** Kari Guderud, Line H. Sunde, Siri T. Flåm, Marthe T. Mæhlen, Maria D. Mjaavatten, Ellen S. Norli, Ida M. Evenrød, Bettina K. Andreassen, Sören Franzenburg, Andre Franke, Simon Rayner, Kristina Gervin, Benedicte A. Lie

**Affiliations:** ^1^ Department of Medical Genetics, University of Oslo and Oslo University Hospital, Oslo, Norway; ^2^ K. G. Jebsen Inflammation Research Centre, University of Oslo, Oslo, Norway; ^3^ Department of Rheumatology, Diakonhjemmet Hospital, Oslo, Norway; ^4^ Department of Rheumatology, Martina Hansens Hospital, Bærum, Norway; ^5^ Department of Research, Cancer Registry of Norway, Institute for Population-Based Research, Oslo, Norway; ^6^ Institute of Clinical Molecular Biology, Christian-Albrechts-University of Kiel, Kiel, Germany; ^7^ Pharmacoepidemiology and Drug Safety Research Group, Department of Pharmacy, University of Oslo, Oslo, Norway; ^8^ PharmaTox Strategic Research Initiative, Faculty of Mathematics and Natural Sciences, University of Oslo, Oslo, Norway; ^9^ Division of Clinical Neuroscience, Department of Research and Innovation, Oslo University Hospital, Oslo, Norway

**Keywords:** Rheumatoid arthritis, T cells, CD4 memory, CD4 naïve, DNA methylation, RRBS, epigenetics, methotrexate

## Abstract

**Background:**

Methotrexate (MTX) is the first line treatment of rheumatoid arthritis (RA), and methylation changes in bulk T cells have been reported after treatment with MTX. We have investigated cell-type specific DNA methylation changes across the genome in naïve and memory CD4^+^ T cells before and after MTX treatment of RA patients. DNA methylation profiles of newly diagnosed RA patients (N=9) were assessed by reduced representation bisulfite sequencing.

**Results:**

We found that MTX treatment significantly influenced DNA methylation levels at multiple CpG sites in both cell populations. Interestingly, we identified differentially methylated sites annotated to two genes; *TRIM15* and *SORC2*, previously reported to predict treatment outcome in RA patients when measured in bulk T cells. Furthermore, several of the genes, including *STAT3*, annotated to the significant CpG sites are relevant for RA susceptibility or the action of MTX.

**Conclusion:**

We detected CpG sites that were associated with MTX treatment in CD4^+^ naïve and memory T cells isolated from RA patients. Several of these sites overlap genetic regions previously associated with RA risk and MTX treatment outcome.

## Introduction

Rheumatoid arthritis (RA) is an autoimmune inflammatory disease leading to joint destruction, disability, systemic complications, shorter life expectancy and increased socioeconomic costs. RA can be divided into two major groups based on the presence or absence of antibodies to citrullinated peptide antigens (ACPA) (1). The RA incidence in Norway is 25 per 100 000 per year (2), and the majority of these patients will start treatment with one or more disease-modifying antirheumatic drugs (DMARDs), with the most extensively used being methotrexate (MTX) (2). The mechanisms of action of MTX are not fully understood, but are shown to involve reduced cell proliferation, increased apoptosis of T cells, increased endogenous adenosine release, altered expression of cellular adhesion molecules, changes in cytokine production, humoral responses and bone formation (3). Recently, the low-dose MTX modulation of inflammatory conditions was suggested to act through suppression of JAK/STAT signaling pathways (4). MTX shows good efficacy in a proportion of patients, but beyond two years after starting MTX, a study showed that only 53-71% of RA patients in close clinical follow-up remained on MTX monotherapy (5). A substantial number of patients do not respond adequately to MTX alone, and it is necessary to either discontinue or combine MTX with other drugs to achieve remission. The consequence of suboptimal treatment and delayed remission might be irreversible joint damage (6). Therefore, a treat-to-target approach, consisting of frequent clinical consultations and a quicker introduction of more potent drugs to reduce the disease activity, has been introduced (7). Additionally, biomarkers to predict treatment response is warranted, since the effect of first treatment is strongly associated with long-term outcome in RA patients (8).

The heterogeneity of the underlying biological mechanisms involved in RA is likely influencing the variable treatment outcome. RA is caused by a complex, and yet poorly understood, interplay between genetic, epigenetic and environmental factors. Smoking is, as of yet, the most important environmental risk factor (9, 10), and more than 100 different loci with single nucleotide polymorphisms (SNPs) alleles associated with increased risk of RA (11). Genetic variants have also been reported to be associated with MTX treatment (12) and MTX response in RA (13, 14) and juvenile idiopathic arthritis (JIA) (15).

Already in 1996, Kim et al. showed that inflammatory arthritis was associated with a significant degree of global DNA hypomethylation in peripheral blood mononuclear cells (PBMC) that was reversed with MTX treatment (16). In 2015, global DNA hypomethylation, which was reversed with MTX, was confirmed in bulk T cells from patients with RA (17). However, irrespective of disease, the overall degree of DNA methylation differs between T cell subsets, and a global loss of DNA methylation naturally occurs during differentiation of CD4^+^ memory cells from CD4^+^ naïve T cells (18). Furthermore, even though global DNA methylation changes have been reported during MTX treatment in RA patients, no specific genomic regions or loci have been identified yet. However, at diagnosis, DNA methylation levels at 21 CpGs, assessed in T cells using Infinium 450k array, have been reported to be associated with subsequent response to diverse DMARDs, and 1/3 of these patients received MTX monotherapy (19).

In the current study, we aimed to reveal genetic regions where DNA methylation is influenced by MTX treatment in T cell subsets from RA patients. To do this, we conducted an epigenome-wide association study using reduced representation bisulfite sequencing (mRRBS) in naïve and memory CD4^+^ T cells isolated from newly diagnosed RA patients before and after MTX treatment to identify differentially methylated positions (DMPs).

## Material and methods

### Subjects

The cohort consists of nine RA patients diagnosed according to the 2010 American College of Rheumatology/European League Against Rheumatism (ACR/EULAR) RA classification criteria (20). Eight patients were included at Diakonhjemmet hospital and one at Martina Hansen’s Hospital between 2014 and 2016 in a clinical cohort; The Norwegian Very Early Arthritis Clinic (NOR-VEAC) observational study (ISRCTN05526276) (21). All RA patients were recruited at the day of diagnosis (newly diagnosed), followed by blood sampling and CD4 T cells isolation on the same day. The patients had not been treated with any DMARDs nor steroids at baseline.

### Clinical Data

All patients were clinically examined and gave a blood sample at two timepoints, baseline and a follow-up. The timepoint for the follow-up was three months for N=6 patients and six months for N=3 patients, and they were all clinically examined by a rheumatologist at the time of blood sample withdrawal. The patients’ disease activity was monitored with Disease activity score 28 DAS28 (22), a composite score which includes the clinical assessment of 28 joints, blood values of inflammatory parameters and a score (0–100) from both the clinician and the patient. Depending on the values, the patients were categorized; >5.1: high disease activity, >3.2, but ≤ 5.1: moderate disease activity and ≤ 3.2: low disease activity. If the patient had DAS28 ≤ 2.6, clinical remission was achieved. The EULAR response criteria were used to classify the patients as non-, moderate or good responders to MTX, depending on change and level of the patients’ DAS28 score (23). DAS28 and EULAR response criteria are used to monitor response to treatment and in this study, the disease progression between the two blood withdrawals.

Available clinical data included 28 and 32 swollen joint count, 28 and 32 tender joint count, patient global health, DAS28, rheumatoid factor-status, ACPA-status, c-reactive protein (CRP), erythrocyte sedimentation rate (ESR), gender, age, smoking status and medication (**Table 1**) at both time points. Radiographs of hands and feet were taken at baseline and follow-up.

**Table 1:** Patient characteristics at baseline and follow-up (months).

*Patient*	*Time of blood sample*	*Gender*	*Age*	*Smoking status*	*RF*	*ACPA*	*CRP*	*ESR*	*TJC 28/32*	*SJC 28/32*	*Pat GA*	*Phys GA*	*MTX*	*DAS28*	*DAS28 change*	*DAS28 Response*	*Disease activity*
*A*	*Baseline*	*Male*	*50*	*Earlier*	*15*	*103*	*2*	*17*	*13/17*	*14/16*	*89*	*40*		*6.3*			*High*
	*Follow-up (3)*		*51*				*1*	*10*	*5/5*	*5/5*	*50*	*40*	*25*	*4.2*	*-2.1*	*Moderate*	*Moderate*
*B*	*Baseline*	*Female*	*40*	*Never*	*37*	*124*	*4*	*19*	*3/3*	*3/4*	*10*	*18*		*3.7*			*Moderate*
	*Follow-up (3)*		*40*				*3*	*6*	*1/3*	*0/1*	*0*	*2*	*20*	*2.2*	*-1.4*	*Good*	*Remission*
*C*	*Baseline*	*Male*	*58*	*Earlier*	*25*	*191*	*17*	*19*	*10/14*	*9/11*	*85*	*70*		*5.9*			*High*
	*Follow-up (3)*		*58*				*2*	*4*	*0/0*	*0/0*	*8*	*4*	*20*	*1.1*	*-4.8*	*Good*	*Remission*
*D*	*Baseline*	*Male*	*73*	*Earlier*	*107*	*>340*	*21*	*45*	*6/6*	*3/3*	*61*	*35*		*5.4*			*High*
	*Follow-up (3)*		*73*				*1*	*7*	*7/7*	*0/0*	*9*	*13*	*20*	*3.0*	*-2.4*	*Good*	*Low*
*E*	*Baseline*	*Female*	*68*	*Earlier*	*32*	*>340*	*9*	*48*	*9/11*	*9/11*	*71*	*46*		*6.2*			*High*
	*Follow-up (6)*		*69*				*3*	*32*	*14/16*	*1/1*	*63*	*40*	*15*	*5.7*	*-0.5*	*No improvement*	*High*
*F*	*Baseline*	*Female*	*73*	*Never*	*21*	*2*	*108*	*68*	*27/29*	*27/29*	*99*	*98*		*8.7*			*High*
	*Follow-up (3)*		*73*				*6*	*15*	*0/0*	*0/0*	*57*		*20*	*2.7*	*-6.0*	*Good*	*Low*
*G*	*Baseline*	*Female*	*57*	*Present*	*7*	*310*	*14*	*44*	*3/3*	*4/4*	*30*	*50*		*4.6*			*Moderate*
	*Follow-up (6)*		*58*				*1*	*6*	*0/0*	*0/0*	*41*	*10*	*15*	*1.8*	*-2.8*	*Good*	*Remission*
*H*	*Baseline*	*Female*	*60*	*Earlier*	*86*	*142*	*17*	*41*	*4/4*	*3/4*	*34*	*40*		*4.7*			*Moderate*
	*Follow-up (3)*		*60*				*4*	*26*	*1/2*	*0/0*	*22*	*15*	*20*	*3.2*	*-1.5*	*Good*	*Low*
*I*	*Baseline*	*Female*	*50*	*Earlier*	*>300*	*162*	*8*	*18*	*1/1*	*1/1*	*45*	*30*		*3.5*			*Moderate*
	*Follow-up (6)*		*51*				*1*	*10*	*0/0*	*0/0*	*7*	*2*	*20*	*1.7*	*-1.8*	*Good*	*Remission*

Time of blood sample, number of months in brackets; RF, rheumatoid factor (IgM); U/mL. Positive ≥25; ACPA, Anti-citrullinated protein antibodies; U/mL. Positive ≥10; CRP, C-reactive protein; mg/L; ESR, Erythrocyte sedimentation rate; TJC, Tender joint count; SJC, Swollen joint count; Pat GA, Patient global assessment. Score 0-100; Phys GA, Physician global assessment. Score 0-100; MTX, Methotrexate; mg; DAS28, Disease activity score with 28 joint count; Disease activity, DAS28 >5.1; High; >3.2 but ≤5.1; Moderate; ≤3.2; Low; ≤2.6 Remission.

### Isolation of Immune Cells and DNA Extraction

The isolation of T cells from all samples was initiated within 30 minutes after blood sampling. 200 ml whole blood was collected using a blood bag (Fresenius Kabi, Oslo, Norway) pre-filled with 2 ml 0.5M EDTA (Thermo Fisher Scientific Inc, Massachusetts, USA) and directly diluted in 300 ml PBS (PBS without MgCl, Thermo Fisher Scientific Inc) with 1 ml 1mM EDTA (1 mM) and 10 ml 2% foetal bovine serum (BioNordika, Oslo, Norway). The blood-PBS solution was transferred to 50 ml SepMate™ tubes (STEMcell Technologies, Vancouver, British Columbia, Canada) pre-filled with 14 ml Lymfoprep (Alere, Massachusetts, USA) and centrifuged following the company’s recommendations. The PBMC enriched cell suspension was washed with PBS (0.4% 2 mM EDTA). The CD4^+^ cells were isolated using EasySep™ Human CD4^+^CD25^+^ T Cell Isolation Kit (STEMcell Technologies) with enrichment of CD4^+^ cells, followed by depletion of CD25^+^ cells. The CD4^+^ CD25low cells were separated into CD4^+^ memory (CD8^-^, CD14^-^, CD16^-^, CD19^-^, CD20^-^, CD36^-^, CD56^-^, CD123^-^, TCR gamma/delta, CD66b, glycophorinA, CD25^-^, CD45RO^+^) and CD4^+^ naïve (CD8^-^, CD14^-^, CD16^-^, CD19^-^, CD20^-^, CD36^-^, CD56^-^, CD123^-^, TCR gamma/delta, CD66b, glycophorinA, CD25^-^, CD45RO^-^) T cells (EasySep™ PE Selection Kit and CD45RO-PE (BioLegend, San Diego, USA). Isolated cells were tested for purity and viability by using BD Accuri™ C6 Cytometer (BD Biosciences, New Jersey, USA). We used Fluorescence Minus One to set the gates.

DNA was isolated from CD4^+^ memory and CD4^+^ naïve T cells using RNA/DNA/Protein Purification Plus Kit (Norgen Biotek Corp, Ontario, Canada), and cleaned using QIAamp DNA Micro Kit (Qiagen, Hilden, Germany). The extracted DNA from the CD4^+^ naïve cells was treated with proteinase K and RNase A (Master-Pure Complete DNA & RNA Purification kit, Epicentre) and we performed clean-up using 1.8x Agencourt Ampure XP beads (Thermo Fisher Scientific). The extracted DNA from CD4^+^ memory cells was treated with 10 mg/ml proteinase K (Sigma-Aldrich, Missouri, USA) and cleaned with Genomic DNA Clean & Concentrator Kit (Zymo Research, California, USA). Extracted and Proteinase K-treated DNA from both cell types was quantified and qualified by Qubit 2.0 fluormeter dsDNA HS Assay Kit (Thermo Fisher Scientific Inc) and NanoDrop (Model ND1000, software v3.0.0, Thermo Fisher Scientific Inc).

### Library Preparation for Multiplexed Reduced Representation Bisulfite Sequencing

The DNA extracted from the CD4^+^ naïve cells was prepped for multiplexed reduced representation bisulfite sequencing (mRRBS) on Illumina HiSeq3000 according to the Boyle et al. mRRBS protocol (24), using the Diagenode Premium RRBS kit (Diagenode, Seraing, Belgium) (25). The mRRBS libraries (N=18) were sequenced with indexes from the Diagenode Premium RRBS kit as 50 base pair, single end reads with 20% PhiX spike control and 6 samples per lane.

The CD4^+^ memory cells were prepped for mRRBS on Illumina HiSeq2500 at Institute of Clinical Molecular Biology, Christian-Albrechts-University of Kiel, using an in-house protocol based on Boyle et al. (24) and Gu et al. (26). The 18 libraries were sequenced as 50 base pair single end reads with 10% PhiX spike control and 6 samples per lane.

Achieved Phred quality score was >28 in all reads used from mRRBS sequencing, for both cell populations.

One sample (technical replicate) was sequenced on both platforms (HiSeq 2500 and HiSeq 3000) and was compared to determine how the different sequencing platforms would impact the results. The correlation was 0.9341 for this sample, and hence we found the results to be comparable.

### DNA Methylation Analysis

#### Sequencing Alignment and Quality Control

**Figure 1:**
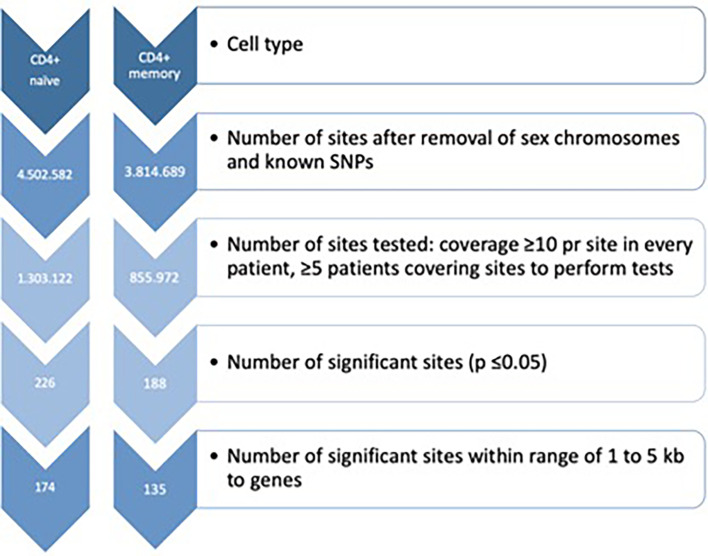
Filtration criteria for identification of differentially DNA methylated CpG sites in both CD4^+^ naïve and memory T cells. SNPs, Single nucleotide polymorphisms.

DNA methylation analyses were carried out using a combination of the programming languages Unix, Python, Java and R (27) with Bioconductor (v2.10) (28). Fastq-files were pre-trimmed and aligned to the current Human Genome version 19 (hg19) with a maximum of two mismatches per read length using BSMAP (29). The *methratio.py* script was used to calculate DNA methylation percentage per loci. Quality control of sequencing reads was initially performed using FastQC (https://www.bioinformatics.babraham.ac.uk/projects/fastqc). After alignment, the alignment efficiency and specificity of the reads were assessed using the *HsMetrics* and *RrbsSummaryMetrics* (Piccard tools, http://broadinstitute.github.io/picard). To reduce the possibility of bias in the analyses, we removed both CpGs on the sex chromosomes and overlapping SNPs, and RnBeads were used for further QC analysis, including manual inspection of the diagnostic QC plots. The list of potentially polymorphic CpGs has been generated using the 2011051 release of the 1,000 Genomes project (30). Computational threshold for inclusion when analysing the mRRBS data were set to include CpGs with ≥ 10 reads to enable identification of minimum 10% difference in DNA methylation. Further, we set the minimum number of samples in each comparison to ≥ 5 samples in both baseline and follow-up samples. To minimize the number of false positives, the data was, in addition to the former filtering, we used false discovery rate (FDR) adjusted p-value ≤ 0.05 (**Figure 1**). Annotation was done using the R package *AnnotatR* (31) from Bioconductor. All genome positions are given according to Genome build 37/hg19. We investigated whether genes annotated to our DMPs were among genes carrying genetic variants involved in MTX treatment efficiency or toxicity (12–15).

#### Differential DNA Methylation

To identify DMPs associated with disease activity and response to MTX, we performed a paired-samples analysis in RnBeads (32). DNA methylation was calculated individually within each patient. Then, baseline DNA methylation was tested against DNA methylation from the follow-up sample individually, and these results were then combined as a mean DNA methylation change across all patients. The regression model was adjusted for known covariates; gender, age, smoking status and ACPA. We performed analyses in naïve and memory CD4^+^ T cells separately. P-values for the DMP level were calculated using the limma method (33) and fitted using an empirical Bayes approach. FDR-adjusted *P*-values ≤0.05 were considered statistically significant. QQ (quantile-quantile) plots with lambda-values and standard errors were generated in the R packages ggplot2 and QQperm.

Overlap between reported RA risk GWAS loci and the significant DMPs observed in our study was investigated using a window size of maximum +/- 500 kb surrounding the SNPs reported by Yarwood et al. (34).

## Results

### Characterization of Patients

All nine patients had active joint inflammation when the baseline blood sample was drawn, and they were all treatment naïve for DMARDs and steroids (**Table 1**), however four patients were taking regular medications for other chronic illnesses (**Supplementary Table S1**). The majority of patients were female (N=6) and ACPA positive (N=8). At baseline, the patients had a mean DAS28 of 5.4, and five patients had high disease activity according to DAS28 (>5.1). Three patients had erosions on radiographs of the hands and/or feet at baseline.

Two patients started treatment with MTX monotherapy, and seven with MTX and prednisolone combined. The MTX start dose was 15 mg/week for all patients, except one who started with 20 mg/week. At follow-up, four patients were in clinical remission (DAS28 ≤2.6) and the mean DAS28 was 2.8. At the time of the second blood sample, the patients either received MTX monotherapy (N=5) or MTX with prednisolone (N=4). Dosage of prednisolone and MTX varied slightly between patients at follow-up (**Table 1** and **Supplementary Table S1**). At follow-up, seven patients had a good response, one patient had a moderate response and one patient had no improvement to MTX according to the EULAR response criteria (23) (**Table 1**).

### Data Generation and Quality Assessment

Flow cytometry showed that the mean purity was 94% (range 85 to 99%) for CD4^+^ memory T cells (CD4^+^CD45RO^+^) and 82% (range 68 to 95%) for CD4^+^ naïve T cells (CD4^+^CD45RA^+^) (**Supplementary Figure S1**). The bisulfite conversion rate was ≥97.2% for CD4^+^ naïve T cells, and ≥99.5% for CD4^+^ memory T cells (**Supplementary Table S2**). The number of aligned mRRBS reads was >13.1 million for each subset of samples (divided by cell type), and the mean CpG coverage was >9.5 per sample (**Supplementary Table S2**). The on target base count was >331 million across the samples, and the mean 10x coverage ranged from 15.9 – 24.3%. Altogether, 1,303,122 and 855,972 CpG sites were investigated in CD4^+^ naïve and CD4^+^ memory T cells, respectively (**Figure 1**). The tested CpGs were generally not covered by the CpGs tested using Infinium 450k or EPIC microarray technologies.

### MTX Treatment Influenced DNA Methylation Levels at Multiple CpG Sites in Both CD4^+^ Naïve and Memory T Cells

**Figure 2:**
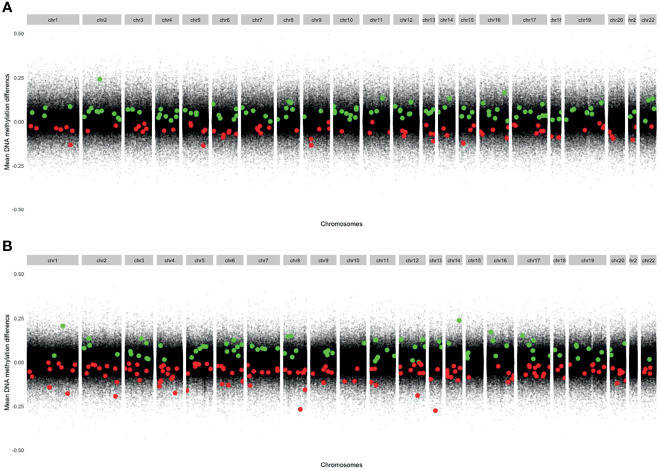
Modified Manhattan plots showing mean DNA methylation differences on the y-axis and genomic positions on the x-axis. Green dots represent significant DMPs with increased DNA methylation at follow-up. Red dots represent significant DMPs with decreased DNA methylation at follow-up. **(A)** CD4^+^ memory T cells (Increased DNA methylation: 111 DMPs. Decreased DNA methylation: 77 DMPs). **(B)** CD4^+^ naïve T cells (Increased DNA methylation: 84 DMPs. Decreased DNA methylation: 142 DMPs). DMPs, Differentially methylated positions.

The mean DNA methylation across all CpGs throughout the genome was higher in CD4^+^ naïve T cells (0.639) than in CD4^+^ memory T cells (0.557) (**Supplementary Table S3**). The distribution of the significant (P_FDR adj <_0.05) DMPs across chromosomes is shown in **Figure 2**, and information about the sites is found in **Supplementary Table S4**. Q-Q plots showed a deviation of the observed from the expected, providing evidence of DNA methylation differences before and after MTX treatment (**Supplementary Figure S2**), but with low inflation (lambda=1.06 for CD4^+^ naïve and lambda=0.84 for CD4^+^ memory T cells).

In CD4^+^ naïve T cells, MTX treatment was associated with 226 significant DMPs, whereas 142 DMPs (62.8%) showed decreased DNA methylation. The 226 DMPs were annotated to 174 genes (**Figure 1**), and the genes for the most significant DMP (P_FDR adj <_5x10^-5^) were *ZNF793*, *DNAAFS*, *TRIM15*, *TRIM10* and *USP37* (**Supplementary Table S4**).

In CD4^+^ memory T cells, MTX treatment was significantly associated with DNA methylation differences at 188 DMPs with 111 DMPs (59.0%) displaying increased DNA methylation. The 188 DMPs represented 135 genes, with *WDR81*, *NXNRD1*, *GRAMD2B*, *TRAF2*, *SPART*, *HDAC4* and *NR2E1* annotated to the most significant DMPs (**Supplementary Table S4**).

Altogether, 593,509 sites were tested in both cell types, but none of the significant DMPs overlapped. However, one gene, *PCDH17*, was found to harbor significant DMPs in both CD4^+^ naïve and memory T cells after MTX treatment. In CD4^+^ naïve T cells, the mean DNA methylation value for the *PCDH17* CpG site was decreased from 57.5% to 32.0% after treatment with MTX, while the mean DNA methylation increased from 4.3% to 5.5% in CD4^+^ memory T cells.

**Figure 3:**
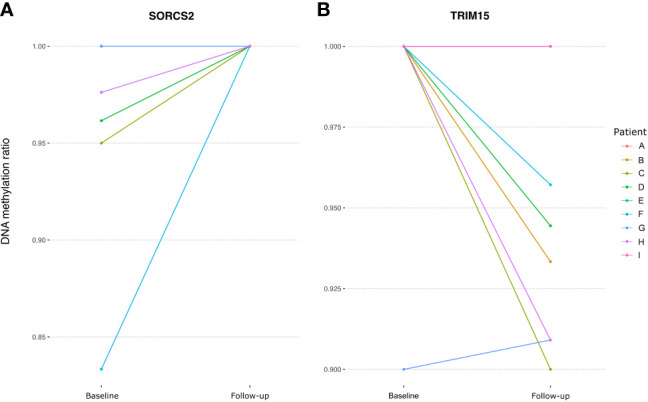
Spaghetti plots showing the change in DNA methylation in RA patients at a DMP located in **(A)** *SORCS2* and **(B)** *TRIM15* between baseline and follow-up. RA; Rheumatoid arthritis, DMP, Differentially methylated position; SORCS2, Sortilin Related VPS10 Domain Containing Receptor 2; TRIM15, Tripartite Motif Containing 15.

Next, we investigated the DNA methylation at genes annotated to the 21 DMPs identified by Glossop et al. to be differentially methylated in T cells collected before treatment from responders vs non-responders (19). Interestingly, two of the genes, *SORCS2* and *TRIM15*, were found to harbor CpGs that significantly changed their methylation levels after MTX treatment. The intronic CpG in *SORCS2* showed variable mean DNA methylation before treatment (96.5%), but was completely methylated in CD4^+^ memory T cells after MTX treatment (**Figure 3A**). All patients with successful measurements of DNA methylation for *SORCS2* at both time points (N=5) had a good response to MTX and had low disease activity (DAS28 ≤ 3.2) at follow-up, but three of the five patients were not in clinical remission (DAS28 ≤ 2.6) (**Table 1**). The CpG located in the promoter of *TRIM15* showed a mean decrease in DNA methylation in CD4^+^ naïve T cells from 98.9% before treatment to 95.0% after MTX. Seven of the nine patients had measures at the CpG in *TRIM15* at both time points (**Figure 3B**). All the seven patients had a good response to MTX and low disease activity (DAS28 ≤ 3.2) after treatment (**Table 1**). Two patients did not show individual decrease in DNA methylation before and after MTX. These two patients were among the three patients who achieved remission (DAS28 ≤ 2.6) at the follow-up time point. The remaining four patients with decrease in DNA methylation did not achieve clinical remission (**Table 1**).

### DMPs Associated With MTX Treatment That Overlap With Genetic RA Risk Loci

**Figure 4:**
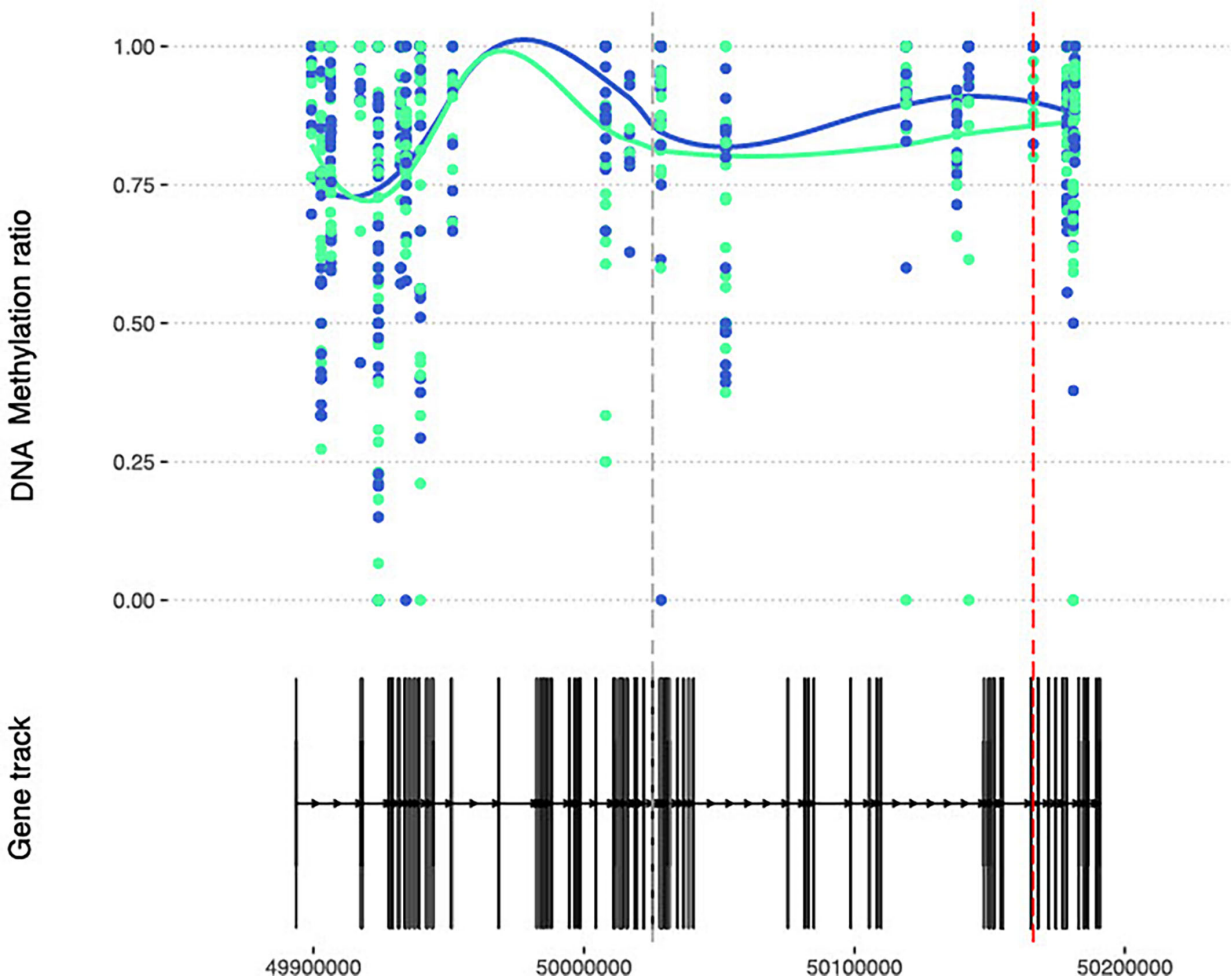
DNA methylation at CpGs overlapping the RA susceptible *WDFY4* gene. DNA methylation at CpGs (N=41) overlapping the *WDFY4* gene. Smoothed lines show mean DNA methylation values for the two patient sampling time points (baseline methylation values in green and follow-up values in blue). The red dotted vertical line marks the position of the DMP, and the grey dotted line marks the RA risk SNP. RA, Rheumatoid arthritis; WDFY4, WD Repeat and FYVE Domain-Containing Protein 4; SNP, Single nucleotide polymorphism.

Next, we wanted to investigate if any of the DMPs associated with MTX treatment in RA patients were in regions overlapping genetic RA risk loci (34), since the genes annotated for SNP risk variants already have been implicated in RA susceptibility and pathogenesis. One could envisage that genes influencing disease development (either through methylation changes or as risk SNPs influencing gene expression levels) also could be involved in the MTX treatment process, especially when patients respond to the treatment. Given that few RA risk loci have been fine mapped, we searched for DMPs located +/- 500 kb from the lead RA risk SNP (**Supplementary Table S5**). In CD4^+^ naïve T cells, 11 DMPs were in the proximity of genetic RA risk loci, and for the *SYNGR1* gene, two DMPs were detected. In CD4^+^ memory cells, 16 DMPs were found near RA risk SNPs, and *CCR6* had two DMPs located within the gene. The only instance where the same gene was annotated for both the risk SNP and the DMP, was for the *WDFY4* gene. *WDFY4* has one significant DMP in CD4^+^ memory T cells before and after treatment, and a trend of increased overall mean methylation in the RA patients after treatment with MTX was seen across several sites (**Figure 4**).

### DNA Methylation Changes in Genes Involved in MTX Response

Interestingly, among the genes annotated to significant DMPs were several solute carrier (SLC) transporters (**Supplementary Table S4**). These transporters play a role in transport of substrates across biological membranes, which is important in the absorption of drugs. Changes in DNA methylation after MTX treatment at CpGs annotated to *SLC6A5*, *SLC9B1*, *SLC25A3*, and *SLC29A1* in CD4^+^ naïve T cells, and *SLC30A5* and *SLC38A1* in CD4^+^ memory T cells (**Table 2**). Mainly increased (*SLC6A5*, *SLC25A3*, *SLC30A5* and *SLC38A1*), but also decreased DNA methylation (*SLC9B1* and *SLC29A1*) was observed at these sites after MTX treatment.

**Table 2:** DMPs annotated to genes previously reported to be relevant to MTX function or treatment response in RA or JIA.

MTX relevance	CD4^+^ T cell subtype	Chr	CpG position	Mean methylation		P-value (FDR adj)	Annotated gene
				Baseline	Follow-up		
**SLC genes**							
	Naïve	11	20631810	0.0327	0.0388	0.0007	*SLC6A5*
	Naïve	4	103840647	1.0000	0.9060	0.0006	*SLC9B1*
	Naïve	6	44186687	0.9218	0.7896	0.0004	*SLC29A1*
	Memory	5	68391268	0.9585	0.9896	0.01	*SLC20A5*
	Memory	12	46660724	0.0071	0.0457	0.003	*SLC38A1*
**Genes harbouring SNPs associated with MTX response in RA**							
	Naïve	3	77184757	0.9753	0.9632	0.003	*ROBO2*
	Naïve	6	21941902	1.0000	0.9799	0.0006	*CASC15*
	Memory	10	1454376	0	0.0607	0.04	*ADARB2*
**Genes harbouring SNPs associated with MTX response in JIA**							
	Naïve	5	153856041	0.0750	0.0620	0.0006	*HAND1*
	Naïve	22	40082539	1.0000	0.9433	0.0006	*CACNA1*
	Naïve	7	77649488	0.0511	0.1329	0.0003	*MAGI2*
	Naïve	8	3773745	0.9442	0.9752	0.008	*CSMD1*
	Memory	10	131747160	0.9080	0.9223	0.03	*EBF3*
**JAK/STAT genes**							
	Naïve	17	40540820	0.0753	0.1973	0.0002	*STAT3*

RA, Rheumatoid arthritis; JIA, Juvenile idiopathic arthritis; MTX, Methotrexate; Chr, Chromosome; FDR, False discovery rate; SLC, Solute carrier; JAK, Janus kinase; STAT, Signal transducer and activator of transcription.

We also investigated whether genes implicated through SNPs found to be associated with MTX response in either RA (13, 14) or JIA (15) were among the genes annotated to CpGs showing differential DNA methylation after MTX treatment in the RA patients (**Table 2**). Indeed, DMPs identified in naïve CD4^+^ T cells were annotated to *ROBO2*, *CASC15*, *HAND1*, *CACNA1, MAGI2* and *CSMD1* genes. The DMPs in the two latter genes showed increased DNA methylation, while the first four showed decreased DNA methylation between the two sample points. For the significant DMPs in CD4^+^ memory T cells, only *ADARB2* (P_FDR adj_=0.04) and *EBF3* (P_FDR adj_=0.03) harbored genetic MTX response variants, and they both showed increased DNA methylation.

**Figure 5:**
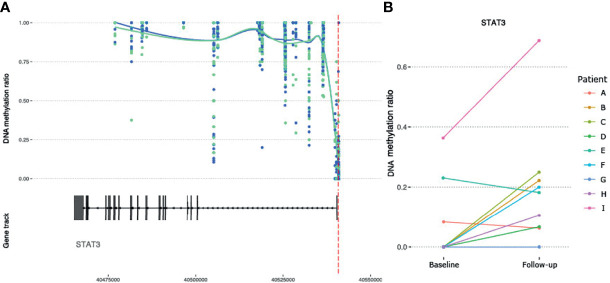
DNA methylation at CpGs overlapping the RA susceptible *STAT3* gene. **(A)** DNA methylation at CpGs (N= 73) overlapping the *STAT3* gene. Smoothed lines show mean DNA methylation values for the two patient sampling time points (baseline methylation values in green and follow-up values in blue). The red dotted vertical line marks the position of the significant DMP. **(B)** Intra-individual changes in DNA methylation between baseline and follow-up for the significant DMP in *STAT3*. RA, Rheumatoid arthritis; STAT3, Signal transducer and activator of transcription 3; DMP, Differentially methylated position.

Given the suggested role of JAK/STAT signaling pathway involved in MTX treatment, as well as a central role in RA (35), we checked whether genes encoding any of the four JAK proteins (*JAK1*, *JAK2*, *JAK3* and *TYK2*) or the seven STAT proteins (*STAT1*, *STAT2*, *STAT3*, *STAT4*, *STAT5A*, *STAT5B* and *STAT6*) were among the genes annotated to the significant DMPs (**Supplementary Table S4**). One DMP was found in the promoter region of *STAT3* (**Table 2** and **Figure 5A**) and showed an overall mean increase in DNA methylation after treatment with MTX in CD4^+^ naïve T cells (**Figure 5B**). Interestingly, in contrast, the two patients with moderate or no improvement in DAS28 after treatment with MTX showed decrease in DNA methylation of *STAT3* after treatment (**Figure 5B**).

## Discussion

By using mRRBS, we identified significant DNA methylation changes associated with MTX treatment, and these changes were distinct in naïve and memory CD4^+^ T cells from RA patients. After MTX treatment, the majority of significant sites showed increased DNA methylation in CD4^+^ memory T cells and decreased DNA methylation in CD4^+^ naïve T cells, compared to the samples drawn from the same patients at the time of diagnosis before DMARD treatment. Some of the DMPs were located in genes earlier described to be differentially methylated in responders and non-responders to MTX in bulk T cells from RA patients before treatment (19), i.e. *TRIM15* and *SORCS2.* In our study, we found that MTX treatment influenced the CpG for *TRIM15* in CD4^+^ naïve T cells, while for *SORCS2* in CD4^+^ memory T cells.

The DNA methylation levels varied largely at the same CpGs between CD4^+^ naïve and memory T cells. Globally, we observed higher methylation in CD4^+^ naïve T cells than in CD4^+^ memory T cells, which is in line with the previously described global loss of DNA methylation in heterochromatic parts of the genome during differentiation from CD4^+^ naïve to memory T cells (18). These findings highlight the importance of investigating DNA methylation profiles for CD4^+^ naïve and memory T cells, separately.

DNA methylation is known to be cell type specific and important for regulation of genes, but the exact mechanism is still unknown. In general, DNA methylation at gene promoter regions is negatively correlated with gene expression, but this is not always the case. Naïve and memory CD4+ T cells have different immune functions and disruption of cell type specific cellular processes as a result of altered DNA methylation may lead to phenotypic alterations. DNA methylation is, in addition and in tight interaction with histone modifications, believed to influence DNA accessibility and binding of e.g. the transcriptional complex including transcription factors and cofactors. Unfortunately, gene expression data is not available for these samples, which makes it difficult to draw any conclusions about the functional consequences of decreased DNA methylation on gene expression and thereby the biological implications for RA.

The design of our study aimed to reduce potential noise in the compared methylation profiles by 1) using a clinically well-described cohort of treatment naïve, newly diagnosed patients, 2) sampling the same patients before and after treatment with MTX, 3) methylation profiling of CD4^+^ naïve and memory T cells, separately, 4) using mRRBS to generate high resolution and genome-wide DNA methylation, and finally, 5) adjusting for gender, age, smoking status and ACPA in the statistical analyses to reduce influence of known covariates on DNA methylation.

This approach, however, reduced the number of patients included. Due to limited cohort size, we could not systematically assess CpGs that could be markers for response to MTX treatment. Future studies regarding treatment response are warranted, and such studies are further motivated by the observation that two of the genes (*TRIM15* and *SORCS2*) with CpGs being influenced by MTX treatment were also reported by Glossop et al. to harbor epigenetic markers for treatment response to several DMARDs (19). Encouragingly in this respect, molecular signatures have been reported to accurately predict anti-TNF treatment response, but with distinct DNA methylation and transcriptomic profiles for RA patients treated with adalimumab and etanercept (36).

A recent study of DNA methylation and gene expression, associated with RA, in bulk CD4^+^ T cells found that 22% of the differentially methylated regions and the differentially expressed genes overlapped the same topologically associated domains (37). A number of these were also linked to genetic RA risk variants through expression or methylation quantitative traits.

The functional relevance of the genes annotated for our significant DMPs is supported by the notion that these genes have been reported to either influence RA risk (34) or in MTX treatment response or pathways (4, 13). Among the genes annotated for DMPs were several SLC transporters, which facilitate the transport of a wide array of substrates across biological membranes, including the absorption of drugs (38).

The most robust methylation differences, i.e. with more than one significant DMP detected, were for *SYNGR1* in CD4^+^ naïve T cells and for *CCR6* in CD4^+^ memory T cells. *SYNGR1* and *CCR6* have been identified as RA susceptibility loci in large GWAS studies (39, 40). Inhibition of ccr6, by monoclonal antibodies in mice, has been reported to supress arthritis (41). We also detected increased DNA methylation after MTX treatment in CD4^+^ memory T cells at a CpG located in the *WDFY4* gene, which is a gene reported to harbour SNPs associated with several rheumatic diseases, i.e. systemic lupus erythematosus, JIA and RA (34, 42–44).

We found an overlap between our DMPs and eight regions harboring SNPs found to predict response to MTX treatment in RA (13, 14) and JIA (15). Our finding of MTX also influencing the DNA methylation at such loci suggest a role for genes in these regions, both genetically and epigenetically, during MTX treatment. Our DMPs draw attention to CD4^+^ naïve and memory T cells being important.

Furthermore, we detected a significant DMP in *STAT3* in CD4^+^ naïve T cells showing increased DNA methylation in RA patients after treatment with MTX, which is of particular interest given the described role of the JAK/STAT pathway in MTX treatment (4). Recently, a study analyzing differential DNA methylation levels in naïve and memory CD4^+^ T cells in RA patients, also detected a central role for JAK1/STAT3/IL6 in these cell subtypes (45). This points to the importance of using anti-IL6 and JAK-inhibitors early in the disease development in RA. Suppression of the JAK/STAT pathway has been suggested to represent one of the principal anti-inflammatory and immunosuppressive mechanism of action of low-dose MTX (46). Expression of the *STAT3* gene has been found to correlate with synovitis and modulation of Th17 differentiation in RA patients (47), while STAT3 inhibition has been reported to mediate chemokine expression in RA synoviocytes (48). A link between STAT3, HIF1α and Notch-1 signalling in regulation of pro-inflammatory mechanisms in RA has also been described (49). Interestingly, when we looked at individual DNA methylation values for *STAT3* in our RA patients, we found that the two patients who obtained moderate or no clinical improvement according to their DAS28 score, showed decreased DNA methylation after treatment with MTX. Obviously, our study is too small to draw any conclusion, but given *STAT3* role as a mediator of inflammatory mechanisms and that methylation changes can alter *STAT3* expression, this is an interesting observation that should be explored further in larger patient cohorts.

## Conclusion

We detected CpG sites with DNA methylation levels associated with MTX treatment in CD4^+^ naïve and memory T cells isolated from RA patients. Several of these CpGs were annotated to genes previously implicated in RA or MTX treatment, including *TRIM15*, *SORCS2*, *CCR6*, *SYNGR1*, *WDFY4*, and in particular *STAT3* given its role of the JAK/STAT pathway in MTX treatment.

## Data Availability Statement

The data presented in the study are deposited in the GEO repository, accession number GSE188509.

## Ethics Statement

The studies involving human participants were reviewed and approved by The National Health Authorities and Ethics Committee (REK #2015/1546 and #2010/719), which was performed in accordance with the Declaration of Helsinki. The patients/participants provided their written informed consent to participate in this study.

## Author Contributions

KGu has contributed with development and during laboratory procedures, developed bioinformatic pipeline, result interpretation and writing. LS has contributed during laboratory procedures, result interpretation and writing of the first draft. SFl has contributed to development and during laboratory procedures. MMæ, MMj, and EN have contributed in recruitment and clinical examination of the patients. IE, SFr, and AF have contributed to the library preparation. BA has contributed to the study design. SR and KGe have contributed to the bioinformatic pipeline and data analyses. BL has contributed to the study supervision, study design, results interpretation and writing. All authors have read, commented and approved the final manuscript.

## Funding

This project was financed by the South-Eastern Norway Regional Health Authority (Helse Sør-Øst).

## Conflict of Interest

The authors declare that the research was conducted in the absence of any commercial or financial relationships that could be construed as a potential conflict of interest.

## Publisher’s Note

All claims expressed in this article are solely those of the authors and do not necessarily represent those of their affiliated organizations, or those of the publisher, the editors and the reviewers. Any product that may be evaluated in this article, or claim that may be made by its manufacturer, is not guaranteed or endorsed by the publisher.

## Abbreviations

ACPA, Anti-citrullinated protein antibody; ACR, American College of Rheumatology; CpGs, CpG sites; CRP, C-reactive protein; DAS28, Disease activity score 28; DMARD, Disease modifying antirheumatic drugs; DMPs, Differentially methylated positions; ESR, Erythrocyte sedimentation rate; EULAR, European League Against Rheumatism; FDR, False discovery rate; GWAS, Genome-wide association studies; JIA, Juvenile idiopathic arthritis; mRRBS, Multiplexed reduced representation bisulfite sequencing; MTX, Methotrexate; NOR-VEAC, The Norwegian Very Early Arthritis Clinic; PBMCs, Peripheral blood mononuclear cells; RA, Rheumatoid arthritis; RF, Rheumatoid factor; SNPs, Single nucleotide polymorphisms.
