# Supplementary material for: Methotrexate Treatment of Newly Diagnosed RA Patients Is Associated With DNA Methylation Differences at Genes Relevant for Disease Pathogenesis and Pharmacological Action
Source: Front Immunol. 2021 Nov 18;12:713611. doi: 10.3389/fimmu.2021.713611 (PMC8637827; doi:10.3389/fimmu.2021.713611)
Supplement: Supplementary file 1 [file DataSheet_1.docx]

A


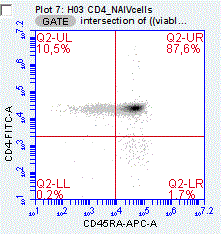


B


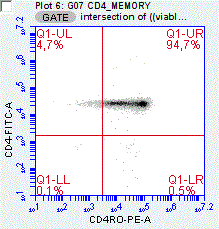


**Supplementary Figure S1:** Representative plots displaying the purity of the sorted cells: A: CD4^+^ naïve T cells. B: CD4^+^ memory T cells

| **Cell type** | **CD4^+^ naïve T cells** | **CD4^+^ memory T cells** |
| --- | --- | --- |
|  | 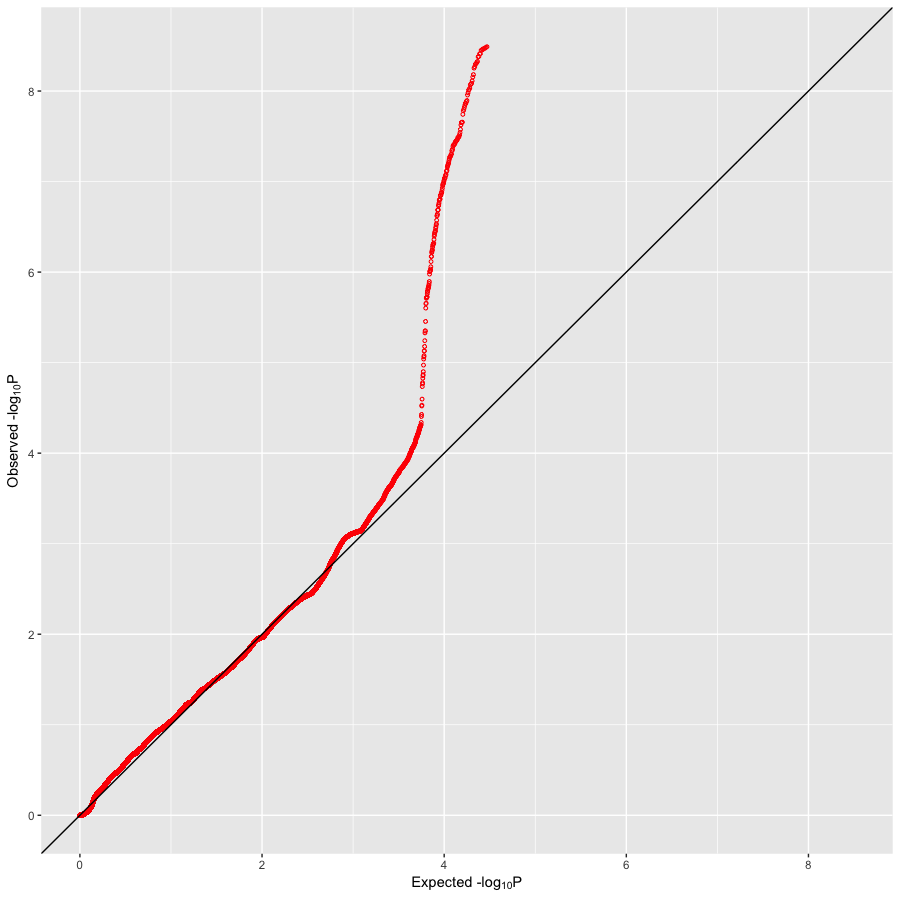 | 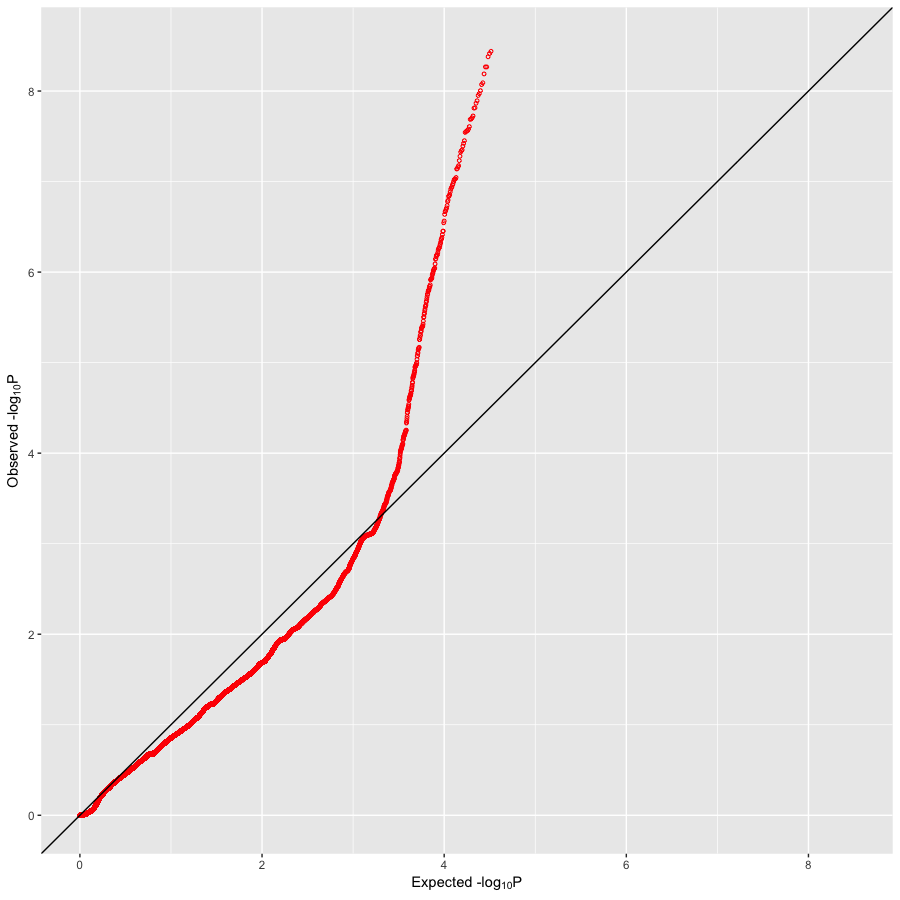 |
| **Lambda (SE)** | 1.0619 (0.0001181792) | 0.8424 (0.0001587648) |

**Supplementary Figure S2:** QQ-plots and lambda values of the association between baseline and floow upin CD4^+^ memory T cells and CD4^+^ naïve T cells

*SE* Standard error
