# Supplementary material for: Methotrexate Treatment of Newly Diagnosed RA Patients Is Associated With DNA Methylation Differences at Genes Relevant for Disease Pathogenesis and Pharmacological Action
Source: Front Immunol. 2021 Nov 18;12:713611. doi: 10.3389/fimmu.2021.713611 (PMC8637827; doi:10.3389/fimmu.2021.713611)
Supplement: Supplementary file 2 [file Table_1.docx]

**Supplementary Table S1:** Medication reported to be taken at the time of sample collection. Follow-up time for each patient in brackets (months).

| \| **Newly diagnosed, treatment naïve RA patients** \| \| \| \| \| \| \| \| --- \| --- \| --- \| --- \| --- \| --- \| --- \| \| **Patient** \| **MTX dosage (mg/week)** \| \| \| **Prednisolone dosage (mg/day)** \| \| **Other medication through the whole period** \| \| **Baseline** \| **Start dosage** \| **Follow-up** \| **Start dosage** \| **Follow-up** \| \| A (3) \| 0 \| 15 \| 25 \| 15 \| 2.5 \|  \| \| B (3) \| 0 \| 20 \| 20 \| 15 \| 0 \| Cetirizine, Ibuprofen \| \| C (3) \| 0 \| 15 \| 20 \| 15 \| 0 \| Naproxen, Ibuprofen \| \| D (3) \| 0 \| 15 \| 20 \| 20 \| 2.5 \|  \| \| E (6) \| 0 \| 15 \| 15 \| 15 \| 0 \| Esomeprazole, Ibuprofen, Paroxetine, Simvastatin, Tapentadol \| \| F (3) \| 0 \| 15 \| 20 \| 20 \| 5 \|  \| \| G (6) \| 0 \| 15 \| 15 \| 0 \| 7.5 \|  \| \| H (3) \| 0 \| 15 \| 20 \| 15 \| 0 \| Diclofenac, Losartan, Paracetamol \| \| I (6) \| 0 \| 15 \| 20 \| 0 \| 0 \|  \| |
| --- | --- | --- | --- | --- | --- | --- | --- | --- | --- | --- | --- | --- | --- | --- | --- | --- | --- | --- | --- | --- | --- | --- | --- | --- | --- | --- | --- | --- | --- | --- | --- | --- | --- | --- | --- | --- | --- | --- | --- | --- | --- | --- | --- | --- | --- | --- | --- | --- | --- | --- | --- | --- | --- | --- | --- | --- | --- | --- | --- | --- | --- | --- | --- | --- | --- | --- | --- | --- | --- | --- | --- | --- | --- | --- | --- | --- | --- | --- | --- | --- | --- | --- |

*RA* Rheumatoid arthritis, *MTX* Methotrexate

**Supplementary Table S2:** Summary of mRRBS performance for CD4^+^ naïve and memory T cell subsets

| **Cell type** | **Time of sampling** | **Reads aligned** | **On target bases** | **Bisulfite conversion** | **1x coverage**  **(%)** | **10x coverage**  **(%)** | **Mean CpG coverage** |
| --- | --- | --- | --- | --- | --- | --- | --- |
| **CD4^+^ naïve T cells** | Baseline | 19,502,452 | 484,450,294 | 97.2 | 49.0 | 24.3 | 12.0 |
| **CD4^+^ naïve T cells** | Follow-up | 20,201,126 | 490,691,572 | 97.5 | 51.0 | 24.0 | 11.7 |
| **CD4^+^ memory T cells** | Baseline | 13,106,830 | 331,691,464 | 99.7 | 40.4 | 15.9 | 9.5 |
| **CD4^+^ memory T cells** | Follow-up | 14,975,456 | 363,665,349 | 99.7 | 42.3 | 17.7 | 10.4 |

CD4^+^ naïve T cells were sequenced on HiSeq 3000, while CD4^+^ memory T cells were sequenced on HiSeq 2500 (see Material and Methods). Data have been generated using HS metrics and mRRBS metrics from Picard tools. Median values across samples are given.

*RA* Rheumatoid arthritis, *MTX* Methotrexate

**Supplementary Table S3:** Global methylation values presented as mean across all CpG sites for each individual. Every patient is presented by two coloumns, representing baseline sampling and follow-up (months).

|  | **CD4^+^ naïve T cells** | | **CD4^+^ memory T cells** | |
| --- | --- | --- | --- | --- |
|  | **Global methylation at baseline** | **Global methylation at follow-up** | **Global methylation at baseline** | **Global methylation at follow-up** |
| A (3) | 0.608 | 0.020 | 0.580 | 0.008 |
| B (3) | 0.662 | 0.002 | 0.584 | -0.062 |
| C (3) | 0.639 | 0.019 | 0.585 | -0.001 |
| D (3) | 0.633 | -0.004 | 0.564 | 0.012 |
| E (6) | 0.639 | 0.014 | 0.542 | 0.071 |
| F (3) | 0.625 | 0.008 | 0.578 | 0.010 |
| G (6) | 0.655 | -0.023 | 0.586 | 0.004 |
| H (3) | 0.623 | -0.003 | 0.586 | 0.021 |
| I (6) | 0.665 | 0 | 0.482 | 0.141 |
| Mean | 0.639 | 0.003 | 0.557 | 0.023 |
